# Supplementary material for: The interactome of intact mitochondria by cross-linking mass spectrometry provides evidence for coexisting respiratory supercomplexes
Source: Mol Cell Proteomics. 2017 Dec 8;17(2):216–32. doi: 10.1074/mcp.RA117.000470 (PMC5795388; doi:10.1074/mcp.RA117.000470)
Supplement: Supplemental Data [file supp_RA117.000470_133922_0_supp_23978_fzffwf.zip › spectra_annotation/mito_spectra_annotation/430-1-10-1-10-1.pdf]

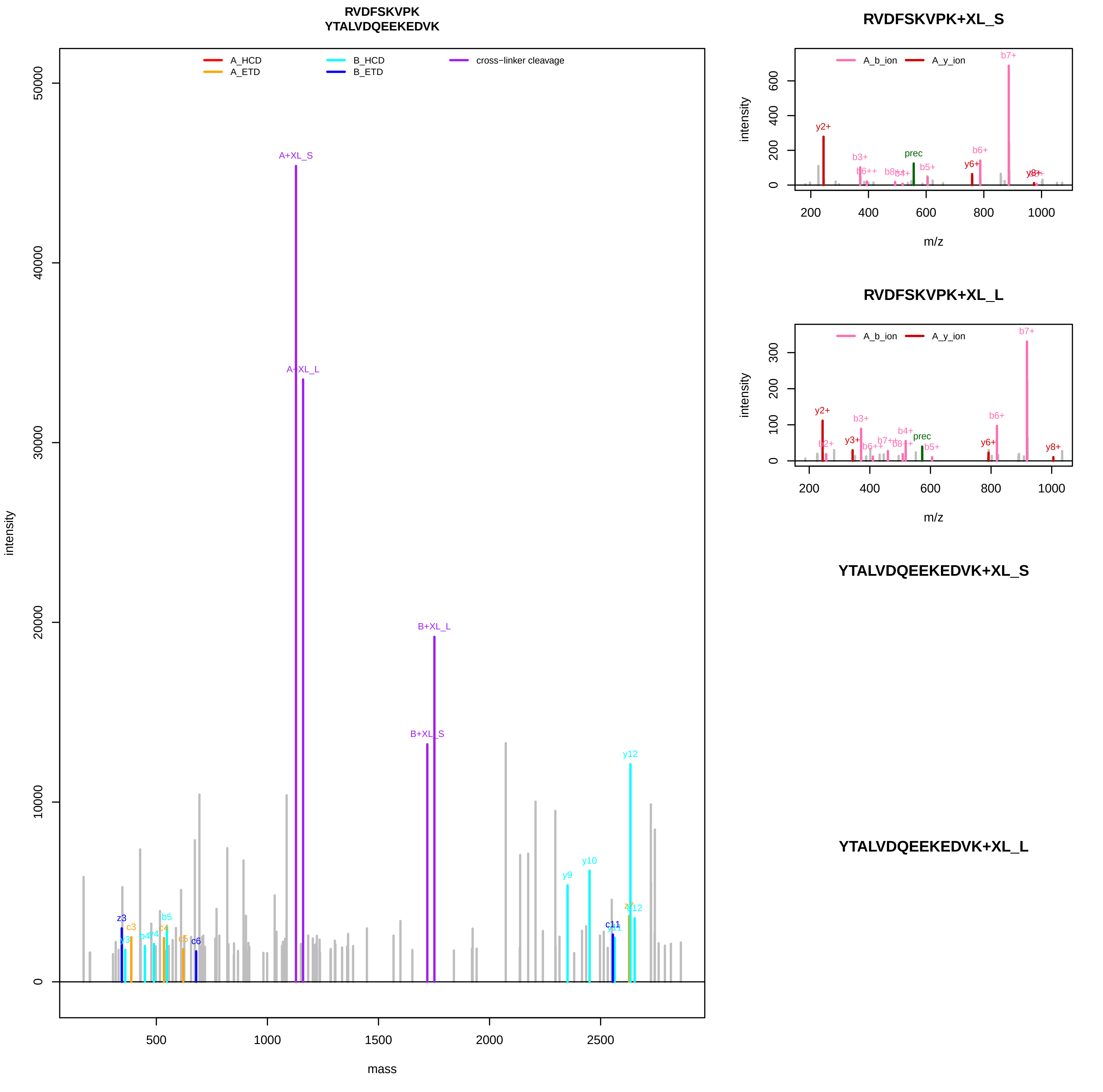

RVDFSKVPK
YTALVDQEEKEDVK
A_HCD
A_ETD
B_HCD
B_ETD
cross−linker cleavage
A+XL_S
A+XL_L
B+XL_L
B+XL_S
y12
y10
y9
c11
z7
y11
z3
c3
y3
b4
y4
b5
c4
c5
c6
intensity
mass
RVDFSKVPK+XL_S
A_b_ion
A_y_ion
y2+
b3+
b6++
b8++
b4+
prec
b5+
y6+
b6+
b7+
y8+
m/z
RVDFSKVPK+XL_L
b2+
y3+
b7++
YTALVDQEEKEDVK+XL_S
YTALVDQEEKEDVK+XL_L
